# Influence of Elytral Color Pattern, Size, and Sex of *Harmonia axyridis* (Coleoptera, Coccinellidae) on Parasite Prevalence and Intensity of *Hesperomyces virescens* (Ascomycota, Laboulbeniales)

**DOI:** 10.3390/insects9020067

**Published:** 2018-06-15

**Authors:** Danny Haelewaters, Thomas Hiller, Michał Gorczak, Donald H. Pfister

**Affiliations:** 1 Department of Organismic and Evolutionary Biology, Faculty of Arts and Sciences, Harvard University, Cambridge, MA 02138, USA; 2 Farlow Reference Library and Herbarium of Cryptogamic Botany, Harvard University, Cambridge, MA 02138, USA; 3 Institute of Evolutionary Ecology and Conservation Genomics, University of Ulm, 89081 Ulm, Germany; 4 Department of Molecular Phylogenetics and Evolution, Faculty of Biology, University of Warsaw, 02-089 Warsaw, Poland

**Keywords:** biotrophic interactions, invasive species, color polymorphism, harlequin ladybird, harmonine

## Abstract

*Harmonia axyridis* is an invasive ladybird (Coleoptera, Coccinellidae) with the potential to outcompete native ladybird species in its invasive distribution area. It was introduced as a biological control agent in many countries but has also spread unintentionally in many others. *Hesperomyces virescens* (Ascomycota, Laboulbeniales) is a minute (200–400 µm in size) biotrophic fungus that infects over 30 species of ladybirds. The aim of this study was to evaluate whether the elytral color pattern, size, and sex of *Ha. axyridis* affect infection by *H. virescens*. Coloration in *Ha. axyridis* has been linked to the presence of an antimicrobial alkaloid (harmonine). In fall 2016, we collected 763 *Ha. axyridis* individuals in Cambridge, Massaschusetts, of which 119 (16%) bore *H. virescens* fruiting bodies. We analyzed 160 individuals (80 infected, 80 uninfected) concerning the intensity of infection by *H. virescens*. Elytral sizes and coloration patterns were quantified using digital photography and analytical methods. Smaller ladybirds had a higher prevalence and higher intensity of parasitism. Additionally, male ladybirds bore more thalli compared to female ladybirds. Elytral color patterns had an effect on neither prevalence nor intensity of infection by Laboulbeniales in our dataset, although we found a slight trend to higher intensity of parasitism in more melanic males. This suggests that the development of Laboulbeniales might be affected by certain insect alkaloids.

## 1. Introduction

The harlequin ladybird *Harmonia axyridis* (Pallas, 1773) (Coccinellidae, Coleoptera) is arguably one of the best-studied and most well-known examples of an invasive insect species. Native to Eastern Asia, it was intentionally introduced, as a biological control agent of aphids and scale insects, first in the USA and later in various European countries. In recent years, however, it has also spread unintentionally in North and South America, Europe, and parts of Asia outside of its native range [1,2]. In Africa, *Ha. axyridis* was introduced intentionally in South Africa (unsuccessful), Egypt, and Tunisia [1]. The recent invasion in South Africa is the result of unintentional introduction [1], whereas the (small) populations in Kenya and Tanzania may represent transient introductions [3,4]. The global invasion of *Ha. axyridis* happened quickly and inspired different facets of research dealing with this species. *Harmonia axyridis* is a major concern, since it causes the displacement of native ladybird species, threatening native ecosystem services [5], and commercial losses in the wine industry [6]. Therefore, efforts are made finding ways to control invasive populations of *Ha. axyridis*, justifying intensive research regarding its natural enemies.

Our group is particularly interested in one of these enemies, *Hesperomyces virescens* Thaxt. (Ascomycota, Laboulbeniales). *Hesperomyces virescens* is a minute (200–400 µm in length) biotrophic fungus that infects over 30 species of ladybirds belonging to 20 genera [7,8]. In recent years, parasite prevalences have increased on *Ha. axyridis*, because this ladybird combines a number of behavioral and life history features that are beneficial for the spread and acquisition of the fungus. Overwintering in large aggregations and a highly promiscuous lifestyle (including males copulating with males) are the most important traits because they allow for many intra- and inter-generational contacts. Like all other Laboulbeniales, *H. virescens* is transmitted nearly exclusively during direct contact between host individuals, especially during mating [9,10]. During feeding/mating season, sexual contacts between hosts result in non-random distribution patterns of the fungus in both sexes (sexual transmission). This contrasts with overwintering multi-layered aggregations of *Ha. axyridis* ladybirds, where direct, non-sexual physical contacts result in randomized infection patterns (social transmission). 

Although delayed in occurrence after the establishment of *Ha. axyridis*, *H. virescens* has been reported from *Ha. axyridis* in most areas of its occurrence. Discovered in the USA in 2002 [11], the *H. virescens*–*Ha. axyridis* combination was later observed in various European and South American countries and South Africa [7,12,13]. In addition, two infected specimens of *Ha. axyridis* collected in China in the 1930s were retrieved during museum collection studies [14]. Due to the charismatic character of its ladybird hosts, their importance in our ecosystems, and the status of its most common host *Ha. axyridis*, *H. virescens* has become one of the best studied species of Laboulbeniales. Seasonal variation of *H. virescens* prevalence was explored in a few publications [9,10,11,15] as well intra- and inter-specific transmission successes [16] and negative effects on its hosts [10,17,18].

In this study we explored the potential link between color polymorphism of *Ha. axyridis* and the prevalence and intensity of *H. virescens* infection. *Harmonia axyridis* is highly polymorphic in color patterns; this polymorphism is controlled by one locus with 15 alleles [19]. The bright, multi-spotted *forma succinea* is usually the most abundant phenotype in North America [1]. However, the proportion of individuals with different phenotypes varies across seasons, mediated by a balance of climate factors, pollution, non-random mating behaviors, and sexual selection [20,21,22,23,24,25]. Moreover, ladybirds of different phenotypes are known to have distinct invasion patterns [26]. Interestingly, the degree of melanization is known to vary depending on environmental conditions, even within the same phenotype [19]. In *forma succinea*, the proportions of black to bright areas (ranging from entirely bright to nearly entirely black) are dependent mostly on temperatures during larval and pupal development. Even though melanic forms of *Ha. axyridis* (*f. axyridis*, *f. conspicua*, *f. spectabilis*) are better adapted to cold conditions [27,28], black coloration is negatively correlated with total alkaloid content, at least in females [29]. Insect alkaloids serve as deterrents against predators but they are also considered to be non-specific defences against pathogens [30]. Here, we aimed to investigate whether color patterns, and thus indirectly alkaloid contents, have an influence on parasitism by *H. virescens*. We hypothesized that both the parasite prevalence and intensity of infection by *H. virescens* are elevated for ladybirds with higher melanic area on their elytra (i.e., with an increased number of black spots and/or increased spot size).

## 2. Materials and Methods 

A total of 763 specimens of *Harmonia axyridis* ladybirds of *forma succinea* were collected from the south and west walls of William James Hall (42.377054 N 71.113421 W), Cambridge, Massachusetts in October–November 2016. All individuals were sexed and screened for infection with *H. virescens* in the laboratory using an Olympus SZX9 stereomicroscope (Olympus, Waltham, MA, USA) at 50×. Infection was determined based on the occurrence of at least one juvenile or adult fruiting body (*thallus*). For every infected ladybird (*n* = 80), we counted all adult thalli present. Maturity was judged by the presence of ascospores within the perithecium. For the intensity of juvenile thalli, we employed the following categories: ‘few’ = 5 juvenile thalli, ‘several’ = 10, ‘some’ = 20, ‘many’ = 30 or more.

Of these ladybirds, we used a balanced dataset of 160 individuals, 80 males (40 infected, 40 uninfected) and 80 females (40 infected, 40 uninfected), for further analyses. For each individual, we measured the area of the left elytron and calculated the percentage of the elytral area covered by black spots. For this purpose, we made images of all 160 left elytra using an Olympus XC50 camera and cellSens Standard 1.14 software (Olympus, Waltham, MA, USA). All images are available for download from the Figshare online repository [31]. To develop an automated method measuring total elytral area and elytral area covered by spots as well as counting the number of spots/left elytron, we designed a macro for the ImageJ platform [32] and coded the script using IJM programming language on Fiji image processing software [33]. The most relevant image processing steps are detailed below.

**Figure 1:**
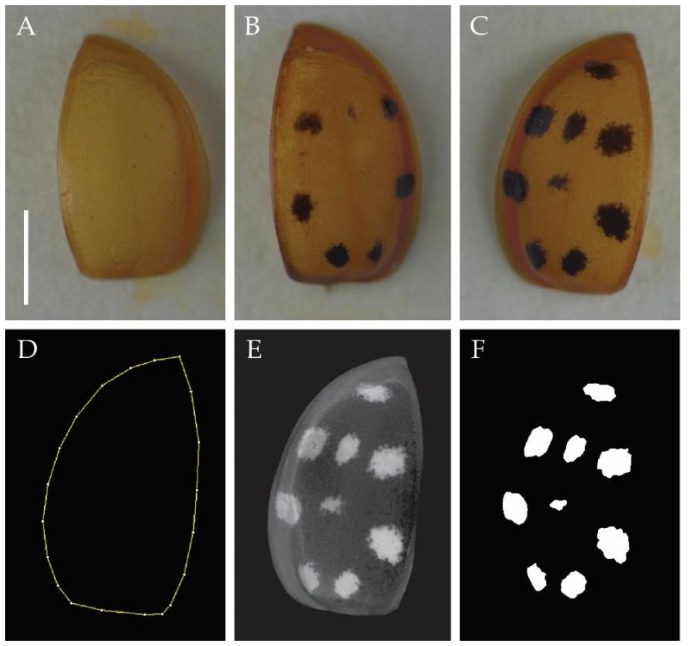
(**A**–**C**) Examples of different elytral sizes and coloration patterns in *Harmonia axyridis forma succinea*. (**D**–**F**) Different image processing steps for the raw image shown in (**C**). (**D**) Left elytron ROI drawn manually using the Polygon tool. (**E**) Modified image after color deconvolution for easy detection of spots. (**F**) Spot ROIs. Scale bar = 2 cm.

For each left elytron image (Figure 1A–C), we manually drew the RIO (Region of Interest) using the Polygon tool in ImageJ (Figure 1D). The macro starts by asking the user to select three folders: a folder with all raw images of left elytra (RGB format, TIFF files), a folder with all left elytron ROIs (_roi.roi files), and a folder for results. Through modification of each RGB image (Colour Deconvolution plugin), the spot regions are detected (Figure 1E) and drawn in binary fashion, as white ROIs on a black background (Figure 1F). The macro then counts the number of detected spots and measures the area of the left elytron ROI and of each spot ROI. The number of spots and measurements are saved in an Excel file in the result folder. The first measurement is the area of the total left elytron ROI, the other measurements (2, 3, 4, etc.) are the areas of the spot ROIs. A single measurement in the resulting Excel file indicates absence of spots. The macro is available for download from Figshare [31].

All statistical analyses were performed in R [34]. We used general linear models (function glm(), R package *stats* [34]) to investigate whether the color pattern has an influence on the prevalence and intensity of parasitism. We used the elytral area and the spot percentage as explaining variables in our models for, first, parasite prevalence (binomial distribution), and, second, thallus count (Poisson distribution with log-link) as response variables. Because the color pattern presented a significant gender bias (Kruskal-Wallis test, function kruskal.test(), R package *stats* [34]), we used the interaction of spot percentage and sex in all our models. We further included the variable sex in our model addressing the thalli count. To compare the total size of males and females, we applied the Student *t*-test (function t.test(), R package *stats* [34]). All continuous variables were standardized to control for differences in magnitude between variables.

We used a likelihood ratio test (function anova() with test=”Chisq”, R package *stats* [34]) to compare our candidate model for each (prevalence and intensity), containing elytral area and spot percentage as explaining variables (Mod1_prev_, Mod1_int_), with the respective Null model (Mod0_prev_, Mod0_int_). Furthermore, we calculated pseudo R2-values to evaluate model fit with the help of R package *sjstats*, using the function r2() [35]. Finally, we used the function plot_model() (R package *sjstats* [35]) to visualize the model results.

## 3. Results

Of the 763 sampled *Ha. axyridis*, 119 individuals were infected with *H. virescens* (parasite prevalence = 16%). We did not find any sex biases in the prevalence, with males (63 out of 423, 15%) being equally likely to be infected as females (56 out of 340, 16%) (*X*^2^ = 0.36, *p* = 0.55). During data exploration, we detected a significant sex bias in the color pattern, with males (mean = 10.6%) having less area of their elytra covered by black spots compared to females (mean = 20.5%) (*H* = 23.4, *p* < 0.001). Interestingly, we did not find differences in total elytral area between males and females (*t* = 0.66, df = 158, *p* = 0.51).

**Figure 2:**
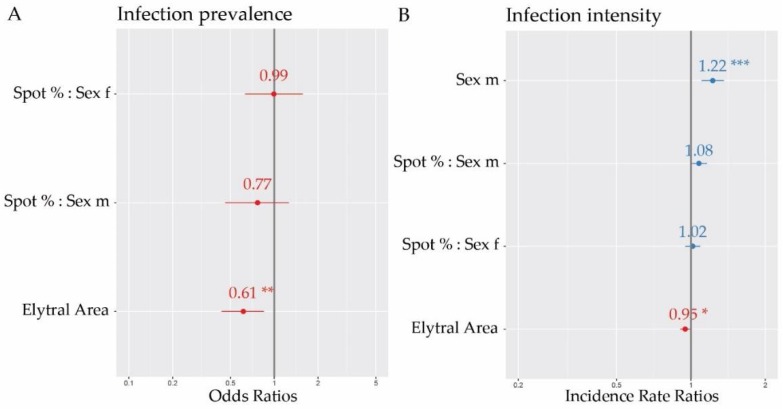
Forest plots representing the results of our modelling approach, showing in (**A**) a significant negative effect of total elytral area on the prevalence of infection of *Ha. axyridis* with *H. virescens* and in (**B**) a significantly higher intensity of parasitism in males compared to females as well as a significant negative effect of total elytral area.

To determine whether color pattern is linked to prevalence and intensity of parasitism with *H. virescens*, we used general linear models. Addressing the parasite prevalence, we included 160 individual beetles, 80 males and 80 females, in our model (Mod1_prev_), which was significantly better than the Null model (Mod0_prev_) (*X*^2^ = 10.4, *p* = 0.03). Although we did not find a link between spot area and parasite prevalence, total elytral area had a strong negative effect, indicating a higher parasite prevalence for smaller elytra (Table 1, Figure 2A). The overall model fit, however, was quite low (Nagelkerke’s *R*-squared = 0.08), suggesting that further variables not included in our model also have an effect on prevalence. 

The intensity of parasitism was measured by counting the number of juvenile and adult thalli on each individual of the 80 infected beetles (40 females and 40 males) and the resulting model (Mod1_int_) was significantly better than the Null model (Mod0_int_) (*X*^2^ = 23.2, *p* < 0.001). We found a strong positive effect of sex of the beetle, with males having significantly more thalli compared to female beetles. Thallus intensity was 20.2 ± 13.9 on females and 24.1 ± 14.0 on males. The elytral area again showed a significant negative effect, indicating more thalli on smaller elytra (Table 2, Figure 2B). Although spot area had no significant effect on intensity of parasitism, we noticed a marginally significant trend for male beetles with higher spot percentages presenting higher numbers of thalli (*p* = 0.0513, Table 2). The overall model fit was medium (Nagelkerke’s *R*-squared = 0.25), suggesting further variables influencing the intensity of parasitism. 

## 4. Discussion

During this study, we collected 763 specimens of the harlequin ladybird *Ha. axyridis* of which 119 (16%) were infected with *H. virescens.* Parasite prevalence did not differ significantly between sexes in our studied population. Whereas previous studies all observed significant differences in parasite prevalence over space and time, there is a trend for male ladybirds to have higher parasite prevalences compared to females [9,15]. This trend is significant in most studies, but in North Carolina there was only significance for one sample (out of four) [10]. The main reason proposed for this outcome is the variation in the number of contacts of females with infected males prior to arriving at aggregation sites during fall (*sensu* [36]). Concordant with previous studies, we found a higher intensity of infection in male ladybirds [9,37]. This has been explained by male mating behavior resulting in a greater chance of contact with other infected individuals. Particularly indiscriminative mating of males with either sex is considered as the major cause for the observed infection patterns [10].

We hypothesized that the color pattern of *Ha. axyridis* influences infection with *H. virescens*. In our dataset of 160 ladybirds, the degree of elytral melanization did not have a significant impact on either the prevalence or intensity of parasitism. However, we did observe a slight trend to higher intensity of parasitism in more melanic males. Bezzerides and colleagues [29] suggested that black coloration is negatively corrected with total alkaloid content. This would indeed imply a positive correlation with parasitism by *H. virescens*. Recently, another type of immune defence was indicated as a potential advantageous innovation of *Ha. axyridis*. Antimicrobial peptides were shown to be potent inducible defences against both bacteria and fungi [38]. Two antibacterial c-type lysosymes augment the two components (harmonine and antimicrobial peptides) of the immune response of *Ha. axyridis* [39]. Because little is known about the infection mechanisms of Laboulbeniales, we do not know what types of immune defence they are prone to. Nevertheless, the *Ha. axyridis*–*H. virescens* system may be an easily available, well-suited model to study these questions.

To our knowledge, this is the first study linking ladybird size to infection by *H. virescens*. Independent of sex, ladybirds with smaller elytral area (and thus smaller body size) were infected more often and with higher intensity. We hypothesize that this might be due to differences in immune response, physical features of the cuticle, or activity between larger and smaller ladybirds. First, small size may be indicative of a weaker immune defence against parasites. Adult body size reflects larval food supply [40]. Indeed, what larval stages receive as nutrition significantly affects the size achieved by adults [41]. Second, it is possible that smaller individuals are more prone to infection by *H. virescens* because of a thinner cuticle that is easier to penetrate [42]. At least for ants (Hymenoptera, Formicidae), a strong correlation between cuticle thickness and body size was found [43]. With regard to Laboulbeniales infections, larger insects with thicker cuticles carry thalli less often than their smaller relatives [29]. Species with thick cuticles are infected primarily on their most vulnerable body parts, where the cuticle is thinnest [30]. For example, most thalli of species in the genus *Herpomyces* Thaxt. are located on the antennae of their cockroach hosts (Blattodea) [42,44].

Our third hypothesis states that smaller ladybirds may be more active during mating, resulting in more physical contacts for the transmission of ascospores. Body size indeed is an important character that has been implicated in affecting competitive capacity, mating success, survival, and other life history traits of organisms [45,46]. For example, studies in the native range of *Ha. axyridis* found that mating males were significantly larger than non-mating ones [22,47]. With regard to reproductive success, the maximum number of eggs laid per day is higher for larger ladybirds (*Coccinella septempunctata* Linnaeus, 1758) compared to smaller ones [*Propylea quatuordecimpunctata* (Linnaeus, 1758)] [48]. On the other hand, no correlation was found between the number of ovarioles and either the body length or body weight of *Ha. axyridis* females [36,49]. Based on the available data from previous studies, it is impossible to support any hypothesis linking ladybird body size with activity (during mating). Focused field and experimental studies are needed to elucidate which factors truly have an impact on this insect/fungus interaction.

## Figures and Tables

**Table 1:** Results for the model addressing the prevalence of infection of *Ha. axyridis* with *H. virescens*.

Explanatory Variable	Estimate	Std. Error	z Value	*p* Value	
(Intercept)	−0.0486	0.1776	−0.2740	0.7843	
Elytral Area	−0.4896	0.1700	−2.8790	0.0040	**
Spot Percentage : Sex f	−0.0061	0.2313	−0.0260	0.9791	
Spot Percentage : Sex m	−0.2642	0.2558	−1.0330	0.3016	

Significance levels at: * < 0.05, ** < 0.01, *** < 0.001.

**Table 2:** Results for the model addressing the intensity of infection of *Ha. axyridis* with *H. virescens*.

Explanatory Variable	Estimate	Std. Error	z Value	*p* Value	
(Intercept)	3.0004	0.0385	77.9380	<2 × 10^−16^	***
Sex m	0.2028	0.0524	3.8690	0.0001	***
Elytral Area	−0.0534	0.0240	−2.2230	0.0262	*
Spot Percentage : Sex f	0.0165	0.0357	0.4620	0.6438	
Spot Percentage : Sex m	0.0745	0.0383	1.9490	0.0513	^(^*^)^

Significance levels at: * < 0.05, ** < 0.01, *** < 0.001; ^(^*^)^ marginally significant.
